# Genetic and biomarker modulation of arterial stiffness change in the SardiNIA population cohort

**DOI:** 10.3389/fepid.2023.1295209

**Published:** 2024-01-04

**Authors:** Nigus G. Asefa, Osorio Meirelles, Edward Lakatta, Edoardo Fiorillo, Angelo Scuteri, Francesco Cucca, Michele Marongiu, Alessandro Delitala, David Schlessinger, Lenore J. Launer

**Affiliations:** ^1^ Laboratory of Epidemiology and Population Sciences, National Institute on Aging, National Institutes of Health, NIH, Baltimore, MD, United States; ^2^ Laboratory of Cardiovascular Sciences, National Institute on Aging Intramural Research Program, NIH, Baltimore, MD, United States; ^3^ Istituto di Ricerca Genetica e Biomedica, Consiglio Nazionale delle Ricerche, Lanusei, Italy; ^4^ Dipartimento Scienze Mediche e Sanita' Pubblica, Universita' di Cagliari, Cagliari, Italy; ^5^ Istituto di Ricerca Genetica e Biomedica (IRGB), Consiglio Nazionale Delle Ricerche (CNR), Cittadella Universitaria di Monserrato, Monserrato, Italy; ^6^ Department of Surgical, Medical and Experimental Sciences, University of Sassari, Sassari, Italy; ^7^ Laboratory of Genetics, National Institute on Aging Intramural Research Program, NIH, Baltimore, MD, United States

**Keywords:** arterial stiffness, longitudinal, pulse wave velocity, genetic variant, lipid, inflammation, environment

## Abstract

**Background and aims:**

Arterial stiffness (AS), quantified by pulse wave velocity (PWV), arises due to impaired arterial elastic tissue and smooth muscle dysfunction. We aimed to examine the longitudinal association of genetic, lipid and inflammation biomarkers with PWV and how these associations may change with aging.

**Materials and methods:**

We utilized genotype and four time-point biomarker data from the SardiNIA cohort [*n* = 6,301; mean baseline age 43.3 (SD 17.3); 58% females]. To investigate the association of PWV with genetic variants, lipid, and inflammation biomarkers, we employed linear mixed modeling, using age as the time scale. Biomarkers exhibiting significant longitudinal associations were categorized into tertiles and individuals within the second tertile or those with heterozygous alleles were excluded, leaving a cohort of 2,000 individuals. This cohort was further divided into four risk groups: low genetic and low biomarker (L-L), low genetic and high biomarker (L-H), high genetic and low biomarker (H-L), and high genetic and high biomarker risk (H-H). Subsequent analyses focused on these risk groups to assess their association to PWV with time.

**Results:**

Using the complete dataset, we found a significant longitudinal association of total cholesterol (TC), triglycerides (TG), fibrinogen (FGN), and total white blood cell count (TWBC) with PWV, all with *p* < 3.33 × 10^−3^. After grouping, individuals with homogeneous risk alleles of SNP rs3742207 and high baseline TG levels (H-H group) exhibited a 1.39-fold higher PWV (m/s) (95% CI, 1.17–1.64, *p = *1.21 × 10^−4^) compared to the L-L group. Similarly, individuals in the H-H group of rs3742207-TWBC combination showed 1.75 times higher PWV (95% CI, 1.48–0.2.07, *p = *1.01 × 10^−10^) compared to the L-L group. Similar patterns were observed for groups based on SNP rs7152623-TWBC risk. Furthermore, these associations became more pronounced with increasing age (*p* < 3.33 × 10^−3^).

**Conclusion:**

The longitudinal association of TG and TWBC biomarkers with PWV varied by SNPs rs3742207 and rs7152623 genotype. Further studies are warranted to investigate the function of genetics, lipids, and inflammation biomarkers on PWV change.

## Introduction

Large arteries are now recognized not merely as passive conduits transporting blood from the heart to the tissues, but rather as active organs playing a crucial role in converting pulsatile flow, originating from the heart, into a continuous flow at the tissue level (1). Arterial stiffness (AS) compromises vascular homeostasis making the aorta and small arteries become less distensible to internal pressure (2, 3). AS is typically assessed using pulse wave velocity (PWV), a non-invasive, simple, and reproducible method. Of several AS diagnosis methods, carotid-femoral (cfPWV) is the most reliable measure and is considered the gold standard (4).

Ageing is the most common non-modifiable risk factor for AS. Data from the Framingham Heart Study has shown that the prevalence of high aortic PWV (>10.7 m/s) was around 1% for individuals younger than 50 years old, but increased to about 38% for those aged between 60 and 69 years old, especially in women (5). Cross-sectional (6–8) and longitudinal (9–11) studies show PWV is an independent risk factor for metabolic and cardiovascular diseases (CVDs), as well as all-cause mortality. By comparison, less is known about the environmental and genetic risk factors that contribute to the adverse effects of AS, and how these may change with ageing (12). However, crosslinking and deposition of extracellular matrix proteins in blood vessels do contribute to the adverse effects, suggesting that intermediary physiologic factors, such as inflammation and lipids, may contribute to, and/or increase as a consequence of PWV. Although there has been no consistency in studies of the specific lipid subtype(s), studies suggest that triglyceride (TG) to high-density lipoprotein (HDL) ratio may increase PWV (13, 14). As for inflammatory conditions or biomarkers, high PWV is more prevalent in individuals with inflammatory conditions, like rheumatoid arthritis (15) as well as high-sensitivity C-reactive protein, fibrinogen, D-dimer, and increasing leukocyte and granulocyte counts (16, 17).

There is relatively little information on the genetic components of AS, but studies suggest PWV has a heritable component that ranges from 35% to 65% in twin studies (18, 19) and from 11% to 40% in family studies (20, 21). In the specific case of the community-based SardiNIA cohort, the narrow-sense heritability of PWV was estimated to be 22.5%, indicating that approximately 23% of the phenotypic variation in PWV can be explained by additive genetic effects (22). A few genes, such as *SLC4A7* (23) *COL4A1* (24) *3'-BCL11B* (25), have been identified through Genome-Wide Association Studies (GWASs). However, these genetic variants account for only approximately 6% of the variance in PWV (26).

Studies have consistently indicated that heritability estimates can differ based on sex and age distributions across various complex phenotypes (27). For example, a twin study investigating PWV revealed a decline in heritability from 62% during visit 1 to 35% during visit 2, accompanied by an increase in the influence of nonshared environmental factors (18), suggests a diminishing contribution of genetic factors to the variation in PWV over time (28). One potential explanation for this inconsistency can be the concept of gene-environment (GxE) interaction, where the impact of genetic variants on PWV might only become apparent under specific environmental conditions, such as an unhealthy lifestyle or elevated levels of PWV-related biomarkers, like lipids and inflammation markers (26). Furthermore, the degree to which an individual's environmental or genetic profile impacts PWV as she/he ages is uncertain. This indicates that it may be useful to investigate whether the impact of environmental and genetic risk factors differs in different age categories.

Currently, there is no evidence to suggest whether an individual's genetic profile affects the association between PWV and lipid and inflammation biomarkers, or if age plays a moderating role. Therefore, in this study we aimed to explore whether the longitudinal relationship of lipid and inflammation biomarkers (serving as proxies for environmental factors) with PWV differs depending on the genetic profiles of individuals. To achieve this, we used PWV, lipid and inflammation biomarkers data gathered at four different time points. Additionally, allelic frequencies of two PWV-associated SNPs identified in our previous study (24) and one from an external study (25) were used.

## Materials and methods

### Study population

**Figure 1:**
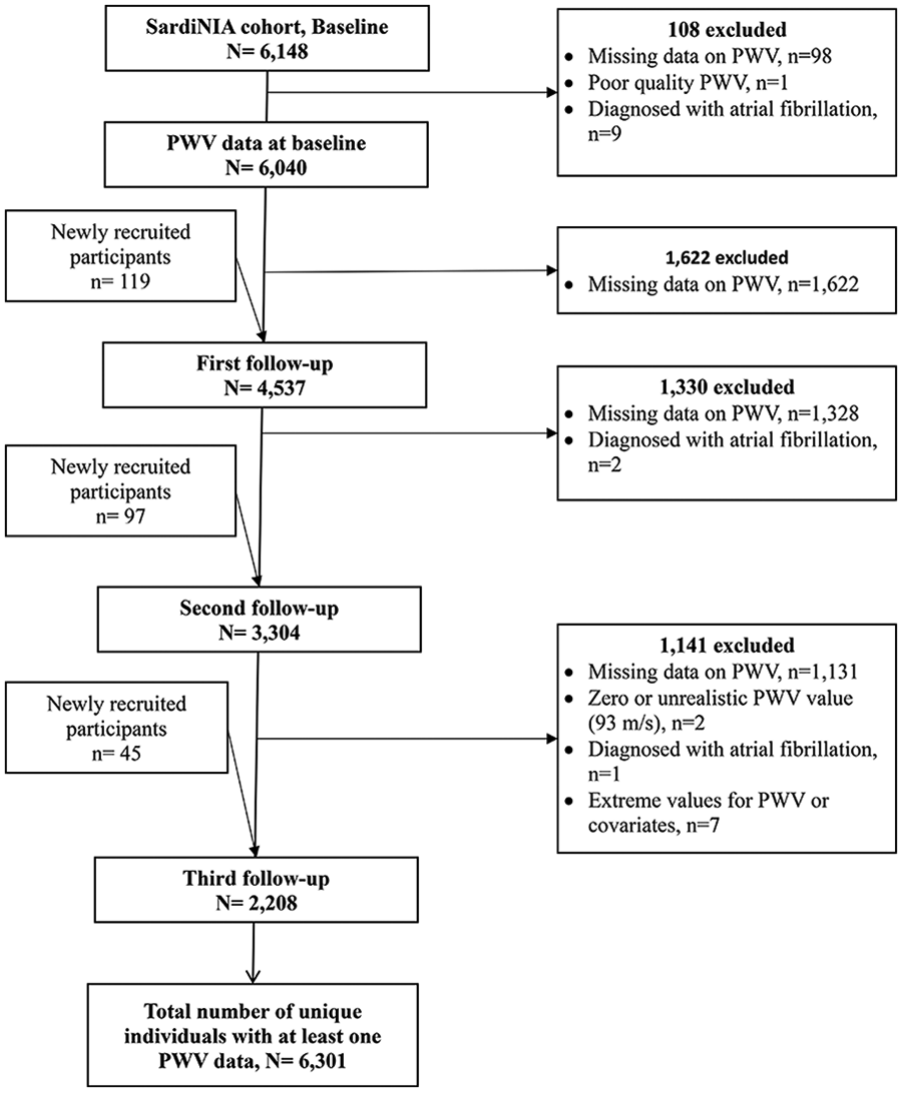
Exclusion criteria and corresponding participant counts at each follow-up time point.

The SardiNIA Study includes baseline data collected between 2001 and 2004, from members of the Sardinian founder population, recruited from a cluster of 4 hill towns above the east coast of the island. The aims of the study, previously described (29), were to investigate the role of genetic and environmental risk factors in complex diseases and traits, including cardiovascular diseases and arterial properties. Briefly, all inhabitants aged 14 years and older were invited via advertisements to participate in a free full health screening. At baseline, 6,148 male and female participants aged 14–102 years were enrolled, representing approximately 60% of the population in the villages (29). Each participant came to the clinic before breakfast, signed consent forms, and gave a sample of fasting blood so that all tests would not be influenced by meals at different times (29). A range of detailed medical history and a full medical examination, including blood pressure (BP), a 12-lead resting electrocardiogram, anthropometric measurements, and measurements of arterial structure and function were taken from each participant (30). A total of 6,301 individuals, aged from 14 years to 101 years, were included in the final analysis of AS. Additional participants were included in the first (*n* = 119), second (*n* = 97), and third (*n* = 45) follow-up exams, resulting in a total of 16,089 observations. However, the number of individuals assessed decreased from 6,040 at baseline to 2,208 at the third follow-up (Figure 1). The median follow-up time between the baseline and the third measurement was 9.8 years [interquartile range (IQR), 9.4–10.1]. The cfPWV, lipids, and inflammatory markers were measured at each exam from baseline to the 3rd follow-up exam using the methods described below.

### Variables measurement

#### Arterial stiffness

The non-invasive measurement of cfPWV is described elsewhere (31). Briefly, ten or more arterial flow waves were recorded from the right carotid artery and the right femoral artery simultaneously using nondirectional transcutaneous Doppler probes (Model 810A, 9–10 Mhz probes, Parks Medical Electronics, Inc, Aloha, OR), and the recorded values were averaged to yield a mean. A custom-designed computer algorithm was employed in order to detect the initial point of systolic flow onset, commonly referred to as the flow's foot. Afterward, a visual examination was conducted by the reader to ensure accuracy and manual adjustments were made as necessary. The time difference between the feet of concurrently recorded carotid and femoral flow waves was then precisely calculated. To determine the distance covered by the flow wave, an external tape measure was utilized along the body's surface. This measurement encompassed the span between the sampling site on the right carotid and the manubrium. The resulting value was then subtracted from the distance between the manubrium and the right femoral sampling site. cfPWV was measured in the same as BP and calculated as the distance traveled by the flow wave divided by the time differential (31).

#### Lipid biomarkers

We examined four time-dependent lipid traits: total cholesterol (TC), triglyceride (TG), high-density lipoprotein cholesterol (HDL), and low-density lipoprotein cholesterol (LDL). The methods used to measure the lipids are described elsewhere (29). In short, blood samples were drawn between 7 and 8 am after overnight fasting. Participants were told not to engage in activities that affect plasma lipids before the collection of samples, e.g., smoking, or physical activity. TG, HDL, and TC were determined by a Colorimetric method (ABA-200 ATC Biochromatic Analyzer; Abbott Laboratories, Irving, TX, USA). High-density lipoprotein cholesterol was determined by a dextran sulfate magnesium precipitation. Low-density lipoprotein cholesterol concentrations were estimated using a newly modified version of Friedewald formula (32).

#### Inflammation biomarkers

We examined eight time-dependent inflammatory markers: fibrinogen (FGN), erythrocyte sedimentation rate (ESR), neutrophils (NEUT), eosinophils (EOS), basophils (BASO), monocytes (MONO), lymphocytes (LYM), and total white blood cells (TWBC) as described by Pilia et al. (22). In short, blood was drawn from each participant and the blood was fractionated to provide, EDTA-plasma, serum, and white and red blood cell subtype counts. Plasma samples were processed using Coulter LH 750 Hematology Analyzer (Beckman Coulter, Inc, CA, USA). FGN concentration was estimated by CoaData Coagulometer 2001/4001 (Helena Biosciences Inc, Sunderland, UK) (22).

#### Genetic data

Briefly, the SardiNIA participants were genotyped using Affymetrix Mapping Arrays. Details of the chips used, imputation of missing genotypes and quality control measures have been detailed elsewhere (24). We followed standard practice to avoid false positives and selected independent SNPs demonstrating genome-wide significance of *p*-value of <5e-8. We also limited our selection to studies that measured cfPWV. We selected two SNPs from our prior GWAS (SNPs rs3742207, located in COL4A1 gene, and rs1495448 located in the MAGI1 gene) (24) and one SNP from external, independent GWAS (SNP rs7152623, located in the 3’ of *BCL11B* gene) (25). Additionally, one SNP meeting the inclusion criteria (23) was excluded because the minor allele frequency was less than 1%. SNPs were extracted using PLINK software (33). Study participants were grouped as high genetic risk if they carried two risk alleles, heterogenous if they carried risk and non-risk alleles, or low risk if homogenous for non-risk alleles.

### Covariates

Covariates were selected based on studies describing associations with PWV (5, 34–39). We included the following covariates at each exam. Body Mass Index {BMI; body weight (kg)/[height (m)]^2^} was based on measured weight and height. Blood pressure (BP) was measured in both arms, after a light breakfast, with a mercury sphygmomanometer using an appropriately sized cuff. Subjects took a 5 min resting period and were in a seated position when their BPs were measured. Values for systolic BP (SBP) and diastolic BP (DBP) were defined by Korotkoff phases I and V, respectively. In the analysis, the average of the second and third measurements on both the right and left arms were used (29). The glucose oxidase method (Beckman Instruments Inc., Fullerton, CA, USA) was employed for the measurement of fasting plasma glucose concentration (29).

Smoking, and drinking status, and medical history, including self-reported use of antihypertensive and lipid-lowering medications were assessed by questionnaire administered at baseline and follow-up exams.

### Data analysis

We included SardiNIA participants who had at least one measure of PWV, between the baseline and the 3rd follow-up. We excluded individuals with poor-quality PWV measures (*n* = 2) or atrial fibrillation (*n* = 12), and 7 individuals with extreme values for the outcome, predictor variables, or covariates.

#### Initial analysis

We used linear mixed modeling with age as the time scale and time-varying independent variables (i.e., each independent variable time point was the same as the PWV time point) to examine the associations of PWV change, separately to each of the above-described lipid and inflammation biomarkers, and SNPs. The mixed models included four time-point observations (*n* = 16,089). Models included a random intercept and random slope and were run using maximum likelihood estimation. Except for models involving genetic variants, which were adjusted only for the effects of sex and age, all other models were adjusted for age, sex, BMI, diastolic BP, fasting plasma glucose, antihypertensive medication, ever smoking, and ever drinking. Additionally, in addition to adjusting for covariates, the models for TC, LDL, and TG were also separately adjusted to account for the effects of HDL.

We normalized the right-skewed PWV with a log transformation. We also used the z-scale transformed data of lipids and inflammation-related biomarkers to ensure that the effect sizes resulting from the linear mixed models could be directly compared across different traits. To enhance the interpretability of the results, we back-transformed the beta-coefficients to their original scale.

#### Interaction analysis

We performed two distinct sets of interaction analyses. First, employing the complete dataset, we conducted interaction analyses involving genetic variants and lipid as well as inflammation biomarkers (Interaction expressed as SNP*Biomarker). We constructed linear mixed model for each biomarker-SNP combination.

Subsequently, to get a clear contrast between individuals with a high vs. low-risk profiles, we selected participants from both the high-risk and low-risk groups of biomarkers and SNPs. We categorized participants into tertiles, based on the baseline levels of biomarkers that were significantly associated with PWV, as determined by the initial analysis. Participants with intermediate biomarker levels (2nd tertiles) and those with heterogeneous alleles were excluded from the interaction analyses. The included individuals (*n* = 2,000) were classified into four groups based on their risk alleles and baseline biomarker levels: *low-low* (L-L, individuals who carried two non-risk alleles of a SNP and who were in the 1st tertile of a biomarker), *low-high* (L-H, individuals carrying non-risk alleles of a SNP and who were in the 3rd tertile), *high-low* (H-L, individuals carrying risk alleles of a SNP and who were in the 1st tertile), and *high-high* (H-H, individuals carrying risk alleles of a SNP and who were in the 3rd tertile). Subsequently, an interaction analysis between these categorized groups (ranging from *n* = 340 to *n* = 656 unique individuals per group) and age was undertaken to investigate whether the strength of the interaction changed with age.

#### Stratified analysis

Previous studies have shown differences between men and women (40) as well as a dramatic increase in PWV after the age of 50 (5). Therefore, using the full dataset, we conducted secondary stratified analyses to examine whether the association between lipids, inflammation-related biomarkers, and genetic variants with PWV differed between individuals under the age of 50 and those over 50, or between the two sexes.

We diagnosed all models to check if they met the assumptions of linear mixed model analysis (i.e., issues related to variance inflation, distribution of residuals, and homoscedasticity). Data cleaning and visualization were carried out using R built-in functions and the tidyverse *R* package, and linear mixed model analysis was implemented using lme4 (41) and the interaction between age and the four groups was tested to ascertain whether PWV differed by group. The significance level of each predictor variable was computed using the lmerTest *R* package. Significance was set at *p *< 0.0033, Bonferroni corrected for 15 model (0.05/15) tests (four lipid and eight inflammation-related biomarkers, and three SNPs).

The present study complies with the Declaration of Helsinki. The locally appointed ethics committee approved the research protocol and informed consent was obtained from the participants (or their legally authorized representative).

## Results

The mean (SD) age (in years) of study participants were, respectively, 43.3 (17.3) and 52.0 (15.1) at baseline and at the third follow-up exam (Table 1). Over the baseline and 3 follow-up studies, the proportion of females remained similar: 58% at baseline and 1st follow-up, and 59% and 60% at the second and third exams respectively. There were relatively (non-significant) more males among those who dropped out, but a slightly higher proportion of smokers dropped out at the 2nd and 3rd follow-ups. BMI levels were largely comparable to those of non-dropouts (Supplementary Table S1).

**Table 1:** Demographic characteristics of the population and longitudinal distribution of lipid, inflammation marker, and genotype data.

Characteristic	Baseline	1st follow-up	2nd follow-up	3rd follow-up
Data collection time	2001–2004	2004–2008	2008–2012	2012–2016
*N*, total	6040	4537	3304	2208
Socio-demography				
Age (years), mean (SD)	43.3 (17.3)	46.2 (16.5)	49.9 (15.9)	52.0 (15.1)
Sex (females), *n* (%)	3480 (58)	2606 (58)	1943 (59)	1323 (60)
Ever smoking (yes)	2242 (37.1)	1822 (40.6)	1340 (40.6)	897 (40.6)
Ever drinking alcohol (yes)	2926 (48.4)	2529 (56.4)	1879 (56.9)	1250 (56.6)
Anthropometry				
Body mass index (kg/m^2^), mean (SD)	25.3 (4.67)	25.5 (4.51)	25.8 (4.53)	25.8 (4.63)
Fasting blood glucose				
Serum glucose level (mg/dl), median (IQR)	85.6 (78.8–93.7)	85.5 (79.4–93.6)	93.0 (86.5–102).	93.0 (84.9–102)
Blood pressure				
Systolic blood pressure (mm Hg), median (IQR)	122 (112–136)	122 (112–134)	122 (112–136)	119 (108–130)
Diastolic blood pressure (mm Hg), median (IQR)	76 (70–84)	78 (70–85)	76.5 (70–83)	72 (66.5–79)
Anti hypertension medication, *n* (%)	603 (10)	572 (12.6)	545 (16.5)	428 (19.4)
Arterial stiffness				
Pulse wave velocity (m/s), median (IQR)	6.14 (5.21–7.57)	6.51 (5.46–8.05)	6.63 (5.63–8.06)	6.94 (5.94–8.841)
Lipid biomarkers				
Total cholesterol (mg/dl), mean (SD)	208 (42.3)	206 (39.0)	216 (39.9)	215 (41.3)
High-density lipoprotein cholesterol (mg/dl), mean (SD)	64.1 (14.9)	63.1 (13.5)	56.9 (14.0)	64.1 (14.3)
Low-density lipoprotein (mg/dl), mean (SD)	128 (36.0)	127 (32.9)	138 (34.2)	132 (35.4)
Triglyceride (mg/dl), median (IQR)	71 (50.2–104)	73.5 (51.9–106)	96.0 (70.6–135)	85.1 (61.3–122)
Inflammation-related biomarkers				
Erythrocyte sedimentation rate (mm/h), median (IQR)	8 (5–14)	5 (3–10)	7 (4–13)	11 (6–19)
Fibrinogen (mg/dl), mean (SD)	329 (67.2)	319 (59.4)	317 (63.3)	335 (68.2)
Neutrophil level (%), mean (SD)	56.6 (8.68)	56.6 (8.45)	57.0 (8.47)	57.3 (9.09)
Eosinophil count (%), median (IQR)	2.2 (1.4–3.3)	2 (1.2–3.2)	2.2 (1.4–3.4)	2.3 (1.5–3.5)
Basophil count (%), median (IQR)	0.3 (0.2–0.4)	0.3 (0.2–0.4)	0.3 (0.2–0.5)	0.4 (0.2–0.5)
Monocyte count (%), median (IQR)	5.7 (4.5–7)	6.1 (4.8–7.6)	6.1 (4.8–7.5)	5.6 (4.2–7)
Lymphocytes count (%), mean (SD)	34.6 (7.90)	34.4 (7.75)	33.7 (7.79)	33.8 (8.22)
Total white blood cell (10^3^/µl), median (IQR)	6.5 (5.5–7.6)	6.5 (5.6–7.6)	6.3 (5.4–7.4)	6.5 (5.5–7.7)
Genotypes				
rs3742207				
TT, *n* (%)	1873 (0.32)	1394 (0.32)	1010 (0.31)	637 (0.30)
GT, *n* (%)	2943 (0.50)	2219 (0.51)	1647 (0.51)	1106 (0.52)
GG, *n* (%)^a^	1091 (0.18)	780 (0.18)	580 (0.18)	389 (0.18)
rs1495448				
GG, *n* (%)	1849 (0.31)	1392 (0.32)	1033 (0.32)	687 (0.32)
TG, *n* (%)	2898 (0.49)	2144 (0.49)	1570 (0.49)	1028 (0.48)
TT, *n* (%)^a^	1160 (0.20)	857 (0.20)	634 (0.20)	417 (0.20)
rs7152623				
GG, *n* (%)	1490 (0.25)	1118 (0.25)	807 (0.25)	528 (0.25)
GA, *n* (%)	2894 (0.49)	2166 (0.49)	1612 (0.50)	1045 (0.49)
AA, *n* (%)^a^	1523 (0.26)	1109 (0.25)	818 (0.25)	559 (0.26)

^a^ Homogenous risk alleles.

### Initial analysis

**Figure 2:**
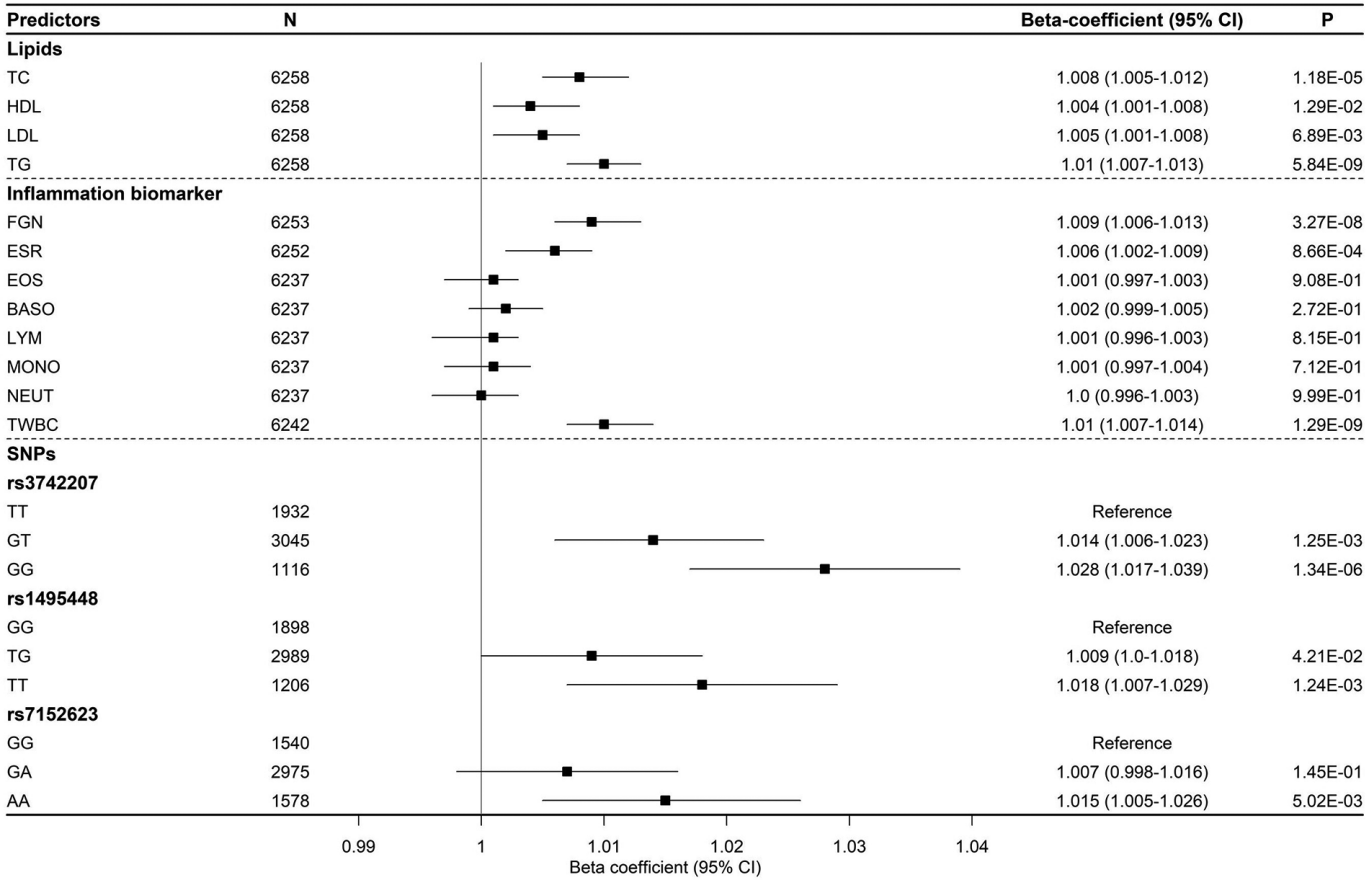
Effect sizes of lipid and inflammation biomarkers, along with genetic variants, on arterial stiffness over time (represented by PWV).

Individuals with elevated lipid profiles had a higher AS. After adjusting for covariates, we observed that an increase of one standard deviation in TC and TG was linked to a rise in PWV (m/s) by 1.008 (95% CI (1.005–1.012), *p = *1.18 × 10^−05^) and 1.01 (95% 1.007–1.013), *p = *5.84 × 10^−09^), respectively (Figure 2). In addition, an increase in one standard deviation of TWBC, ESR, and FGN was associated with a significant increase in AS, with all *P*-values < 0.0033, the *P*-value cutoff for 15 tests. We found a suggestive association of PWV with LDL, but not with any of the white blood cell components (MONO, LYM, NEUT, BASO, or EOS) (see Figure 2).

We observed that individuals with homogeneous risk alleles (GG) had a significantly higher PWV than carriers of the non-risk alleles (TT) for SNP rs3742207 (1.028, 95% CI (1.017–1.039), *p = *1.34 × 10^−06^). Moreover, we found that individuals who carried the AA genotype for SNP rs7152623 had a 1.015 times higher PWV (95% CI: 1.005–1.026, *p = *5.02 × 10^−03^) compared to those with a non-risk allele homozygous genotype (GG) (refer to Figure 2).

### Interaction analysis

Using the full dataset, a suggestive interaction (0.0033 < *P*-Value < 0.05) was observed between SNP rs1495448 and TG, SNP rs1495448 and ESR, and SNP rs7152623 and ESR (Supplementary Table S2). Upon categorizing individuals based on their biomarker and genetic risk levels, we found that the association of lipids (TC, LDL, and TG) and inflammation markers (TWBC, ESR, and ESR) with PWV varied based on the specific genetic variants that these individuals were tested for. For instance, based on rs3742207-TG combination, the H-H group (individuals with homogeneous risk alleles of SNP rs3742207 and high baseline TG) had a significantly higher PWV [beta estimate = 1.39 + 1.59 (age-related interaction effect), *p *< 0.0033] compared to the corresponding L-L group (as shown in Table 2). Likewise, in comparison to individuals within the L-L group of rs3742207-TWBC combination, those falling under the H-H category (individuals carrying risk alleles of SNP rs3742207 and positioned in the 3rd tertile of TWBC) demonstrated elevated PWV levels (1.75, 95% CI: 1.48–2.07, *p = *1.01 × 10^−10^) along with a substantial age-related interaction effect (1.36, 95% CI: 1.16–1.7, *P = *1.40 × 10^–04^).

**Table 2:** The effect of age (Z-scaled) and participants’ group (based on their genetic, lipid, and inflammation biomarker profile) and their interaction on arterial stiffness (represented by PWV).

		SNP rs3742207		SNP rs1495448		SNP rs7152623	
Biomarker	Variables	Estimate (95% CI)	*p*	Estimate (95% CI)	*p*	Estimate (95% CI)	*p*
TC	Age	3.08 (2.76–3.42)	**1.13E-90**	3.01 (2.7–3.37)	**6.00E-82**	2.88 (2.55–3.25)	**1.29E-64**
	L-L	Reference		Reference		Reference	
	L-H	0.88 (0.75–1.02)	9.61E-02	0.91 (0.77–1.07)	2.42E-01	0.96 (0.81–1.13)	5.99E-01
	H-L	1.32 (1.11–1.59)	**2.13E-03**	1.16 (0.97–1.4)	1.09E-01	1.32 (1.12–1.56)	**1.04E-03**
	H-H	1.05 (0.88–1.26)	5.85E-01	1.07 (0.89–1.29)	4.61E-01	1.12 (0.94–1.33)	1.95E-01
	Age × L-H	1.26 (1.08–1.46)	**3.01E-03**	1.32 (1.13–1.54)	**6.04E-04**	1.45 (1.23–1.72)	**1.64E-05**
	Age × H-L	1.14 (0.96–1.34)	1.28E-01	1.14 (0.97–1.35)	1.20E-01	1.14 (0.98–1.33)	9.15E-02
	Age × H-H	1.5 (1.26–1.79)	**5.05E-06**	1.37 (1.15–1.64)	**5.53E-04**	1.55 (1.32–1.83)	**1.83E-07**
LDL	Age	3.1 (2.79–3.44)	**9.06E-94**	3.23 (2.91–3.6)	**1.21E-98**	3.15 (2.81–3.53)	**3.22E-82**
	L-L	Reference		Reference		Reference	
	L-H	0.85 (0.73–0.99)	**3.90E-02**	0.87 (0.74–1.02)	8.43E-02	0.94 (0.79–1.11)	4.47E-01
	H-L	1.34 (1.12–1.6)	**1.33E-03**	1.17 (0.98–1.4)	8.62E-02	1.34 (1.14–1.58)	**4.02E-04**
	H-H	1.08 (0.9–1.29)	4.19E-01	0.99 (0.83–1.19)	9.19E-01	1.07 (0.9–1.26)	4.59E-01
	Age × L-H	1.25 (1.07–1.45)	**4.19E-03**	1.25 (1.08–1.46)	**3.81E-03**	1.27 (1.08–1.5)	**4.46E-03**
	Age × H-L	1.14 (0.96–1.34)	1.26E-01	1.05 (0.89–1.24)	5.52E-01	1.08 (0.93–1.25)	3.37E-01
	Age × H-H	1.6 (1.34–1.9)	**1.30E-07**	1.24 (1.04–1.48)	**1.79E-02**	1.42 (1.21–1.67)	**1.89E-05**
TG	Age	2.79 (2.52–3.08)	**3.80E-87**	2.98 (2.68–3.31)	**1.77E-87**	3.13 (2.8–3.5)	**8.78E-86**
	L-L	Reference		Reference		Reference	
	L-H	1.06 (0.91–1.23)	4.82E-01	0.92 (0.79–1.08)	3.07E-01	0.93 (0.8–1.09)	3.96E-01
	H-L	1.18 (1–1.39)	5.64E-02	0.89 (0.75–1.06)	1.93E-01	1.09 (0.93–1.28)	2.77E-01
	H-H	1.39 (1.17–1.64)	**1.21E-04**	1.23 (1.03–1.46)	**2.05E-02**	1.08 (0.92–1.27)	3.41E-01
	Age × L-H	1.55 (1.35–1.79)	**1.10E-09**	1.37 (1.19–1.59)	**2.53E-05**	1.31 (1.12–1.53)	**7.44E-04**
	Age × H-L	1 (0.85–1.17)	9.90E-01	0.9 (0.76–1.05)	1.87E-01	0.96 (0.82–1.11)	5.63E-01
	Age × H-H	1.59 (1.36–1.86)	**7.27E-09**	1.62 (1.37–1.9)	**8.64E-09**	1.4 (1.21–1.63)	**1.11E-05**
TWBC	Age	3.23 (2.91–3.57)	**1.11E-107**	3.2 (2.88–3.55)	**1.03E-99**	3.23 (2.91–3.59)	**2.37E-100**
	L-L	Reference		Reference		Reference	
	L-H	1.42 (1.23–1.64)	**2.66E-06**	1.19 (1.03–1.39)	**2.26E-02**	1.13 (0.97–1.32)	1.12E-01
	H-L	1.31 (1.12–1.54)	**8.35E-04**	1.03 (0.88–1.21)	7.21E-01	1.17 (1.01–1.35)	**3.80E-02**
	H-H	1.75 (1.48–2.07)	**1.01E-10**	1.36 (1.15–1.61)	**2.83E-04**	1.42 (1.23–1.65)	**2.61E-06**
	Age × L-H	1.19 (1.04–1.37)	**1.18E-02**	1.12 (0.97–1.29)	1.24E-01	1.03 (0.89–1.19)	7.31E-01
	Age × H-L	1.06 (0.91–1.24)	4.40E-01	1 (0.86–1.17)	9.86E-01	1.03 (0.9–1.19)	6.33E-01
	Age × H-H	1.36 (1.16–1.6)	**1.40E-04**	1.15 (0.98–1.34)	8.09E-02	1.2 (1.04–1.38)	**1.24E-02**
ESR	Age	3.07 (2.78–3.38)	**2.26E-105**	3.03 (2.75–3.33)	**2.26E-110**	2.96 (2.66–3.29)	**1.12E-84**
	L-L						
	L-H	1.13 (0.96–1.31)	1.32E-01	1.22 (1.04–1.43)	**1.37E-02**	1.16 (0.99–1.37)	6.99E-02
	H-L	1.33 (1.15–1.53)	**1.52E-04**	1.21 (1.04–1.4)	**1.25E-02**	1.33 (1.16–1.52)	**2.77E-05**
	H-H	1.23 (1.03–1.46)	**1.96E-02**	1.18 (0.99–1.4)	6.51E-02	1.24 (1.05–1.46)	**9.83E-03**
	Age × L-H	1.23 (1.07–1.4)	**2.88E-03**	1.23 (1.08–1.41)	**1.93E-03**	1.32 (1.14–1.52)	**2.33E-04**
	Age × H-L	1.08 (0.94–1.25)	2.87E-01	0.98 (0.85–1.14)	8.21E-01	1.21 (1.06–1.39)	**5.80E-03**
	Age × H-H	1.46 (1.26–1.7)	**1.03E-06**	1.23 (1.06–1.43)	**5.60E-03**	1.32 (1.14–1.52)	**1.19E-04**
FGN	Age	3.33 (3.03–3.68)	**1.76E-122**	3.19 (2.88–3.53)	**6.08E-104**	3.15 (2.83–3.51)	**1.86E-94**
	L-L	Reference		Reference		Reference	
	L-H	0.95 (0.83–1.08)	4.38E-01	0.9 (0.78–1.04)	1.42E-01	0.98 (0.84–1.13)	7.48E-01
	H-L	1.16 (1–1.35)	**4.48E-02**	1.03 (0.88–1.21)	6.72E-01	1.17 (1.01–1.34)	**3.12E-02**
	H-H	1.19 (1.02–1.4)	**3.09E-02**	1.06 (0.91–1.25)	4.58E-01	1.22 (1.05–1.4)	**8.17E-03**
	Age × L-H	0.93 (0.82–1.06)	2.76E-01	1.04 (0.91–1.2)	5.38E-01	1.08 (0.94–1.25)	2.75E-01
	Age × H-L	1.02 (0.88–1.18)	7.69E-01	0.99 (0.85–1.14)	8.53E-01	1.1 (0.96–1.26)	1.95E-01
	Age × H-H	1.13 (0.97–1.32)	1.07E-01	1.12 (0.96–1.31)	1.37E-01	1.13 (0.99–1.3)	7.87E-02

Linear mixed effects model adjusted for age, sex, body mass index, diastolic blood pressure, ant-hypertension medication, plasma glucose, ever smoking, and ever drinking was used. Each SNP-biomarker pair was modeled separately. SNP, single nucleotide polymorphism; PWV, pulse wave velocity; TC, total cholesterol; LDL, low-density lipoprotein; TG, triglyceride; TWBC, total white blood cells; ESR, erythrocyte sedimentation rate; FGN, fibrinogen.

Group description:

L-L: low-low (individuals carrying homogenous non-risk alleles of a SNP and who were in the 1st tertile of lipid or inflammatory biomarker at baseline).

L-H: low-high (individuals carrying homogenous non-risk alleles of a SNP and who were in the 3rd tertile of lipid or inflammatory biomarker at baseline).

H-L: high-low (individuals carrying homogenous risk alleles of a SNP and who were in the 1st tertile of lipid or inflammatory biomarker at baseline).

H-H: high-high (individuals carrying homogenous risk alleles of a SNP and who were in the 3rd tertile of lipid or inflammatory biomarker at baseline).

Bold text indicates associations with both suggestive (0.0033 < *p*-value < 0.05) and significant (*p*-value < 0.0033) findings.

### Stratified analysis

**Figure 3:**
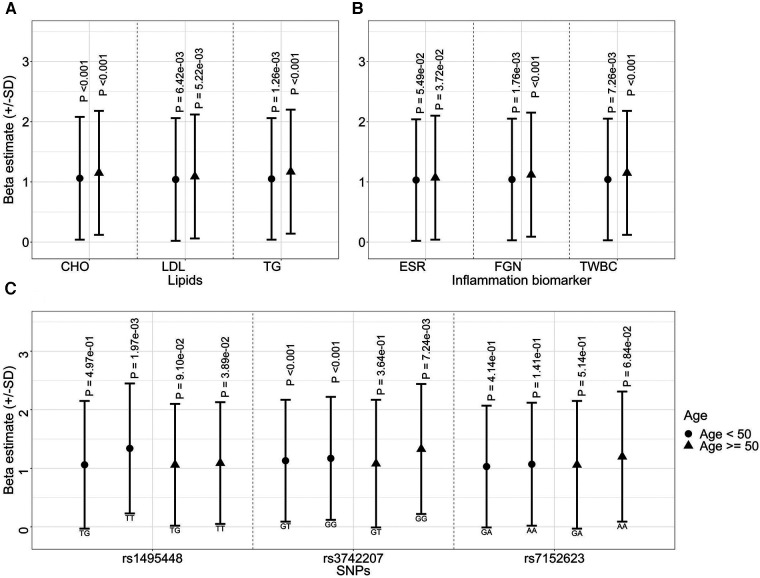
Effect sizes of lipids (**A**), inflammatory biomarkers (**B**), and genetic variants (**C**) on arterial stiffness (represented by PWV) over time, stratified by baseline age.

The longitudinal association of PWV with lipid and inflammation biomarkers remained significant when individuals were stratified by age, with a stronger correlation observed among the older population. Specifically, in individuals under the age of 50, one standard deviation increase in TG was associated with a 1.05 increase in PWV (95% CI: 1.02–1.08, *p = *1.26 × 10^−3^) (Figure 3A). By contrast, for those aged 50 or older, this value increased to 1.17 (95% CI: 1.1–1.24, *p = *5.9 × 10^−07^). A similar pattern of association was observed between PWV and TWBC in the stratified analysis (Figure 3B).

Compared to individuals carrying non-risk alleles of SNPs rs3742207, those carrying homozygous risk alleles exhibited higher PWV values in both age groups. Notably, unlike the lipid or inflammation markers, this effect was more prominent in the younger group (heterozygous, 1.13, 95% CI 1.06–1.196, and homozygous risk allele 1.17, 95% CI 1.08–1.27, both *p *< 0.0033) (Figure 3C). Similarly, the association between SNPs rs1495448 and PWV was observed in both age groups. However, the effect size was more pronounced among the younger cohort.

Moreover, our investigation suggests gender-specific effects of genetic variants and lipids on AS, with no gender differences in inflammation biomarkers (Supplementary Table S3). Specifically, in females, a one standard deviation increase in TG correlated with a 1.1-fold (95% CI: 1.06–1.14, *p = *7.22 × 10^−08^) rise in AS, while in males the values was 1.05 (95% CI: 1.0–1.1, *p = *5.49 × 10^−02^). Additionally, SNPs rs1495448 and rs7152623 displayed significant associations with PWV in males, while such associations were not evident among females (Supplementary Table S3).

## Discussion

We used longitudinal data from the SardiNIA cohort to examine the interaction and relationship between genetic variants and lipid and inflammation biomarkers with AS. We demonstrated the longitudinal association of lipids (TC and TG) and inflammation (ESR, FGN, and TWBC) biomarkers with PWV. Furthermore, our findings indicated that the association of lipid and inflammatory biomarkers with PWV varies based on the allelic frequencies of SNPs rs3742207, rs7152623, and rs1495448. Specifically, PWV was significantly higher among individuals with risk alleles of SNP rs3742207 and high baseline TG and TWBC levels, compared to individuals with non-risk alleles and low baseline TG and TWBC. When stratified by age (<50 vs. 50 or older), we observed significant associations with PWV in both age groups. Notably, biomarkers exhibited larger effect sizes, while genetic variants showed smaller effect sizes among individuals aged 50 and older.

The observed association between PWV and TC and TG in our study is consistent with the findings of two studies based on the UK (Biobank data) (14) and China (42). Furthermore, both studies demonstrated a strong association between TG and AS. However, unlike this study, the above two studies primarily focused on TG/HDL ratio and included relatively older participants without pre-existing cardiovascular disease. Furthermore, we utilized the carotid-femoral measurement for PWV, which differs from the brachial-ankle outcome employed in the study conducted in China (42) as well as from the PWV derived using the pulse waveform collected at the finger in the UK Biobank (14). Taken together, these studies consistently suggest that compared to other lipids, TG may be an important biomarker for the development of AS.

How elevated lipids lead to the development of AS is not yet fully understood. However, a few mechanisms have been suggested, including endothelial dysfunction, the formation of atherosclerotic plaques, and increased vascular extracellular matrix. Over time, these processes can cause structural and functional changes in the vessel wall, leading to the narrowing of the arteries and impeding blood flow. Another suggested mechanism for how elevated TG leads to elevated PWV emphasizes its effect on insulin; high levels of triglycerides can interfere with insulin signaling, making cells less responsive to the hormone (i.e., insulin resistance) (43). The TG-glucose-index, a product of serum TG and glucose levels, is indeed a marker for the assessment of insulin resistance (44, 45). Insulin resistance can cause the endothelial cells to produce less nitric oxide, a molecule that helps to dilate blood vessels and promote arterial flexibility (46).

Notably, TG can also contribute to arterial inflammation. When TG-rich lipoproteins are deposited on the arterial wall, they can damage the endothelium, allowing them to infiltrate into the arterial intima. This process increases the recruitment and attachment of monocytes, which can in turn activate pro-inflammatory signaling pathways, ultimately resulting in arterial wall inflammation (47).

Prior cross-sectional studies have identified significant associations between PWV and ESR (48) FGN (49) and TWBC (17, 50) based on cross-sectional data. Our longitudinal study may serve to validate this existing evidence. On the other hand, while each component of the TWBCs has a distinct inflammatory role and may contribute differently to the development of AS, their lack of association with PWV may suggest that the aggregated effect, i.e., chronic inflammation as the core characteristic, is more important than the specific type of inflammation.

The association between inflammation and arterial stiffness involves complex interplay of cellular and molecular processes. One proposed mechanism is via oxidative stress, with inflammatory cytokines triggering the generation of reactive oxygen species (ROS), leading to damage to biomolecules, proteins, and genes. This damage, can in turn result in endothelial dysfunction and arterial injury, ultimately contributing to arterial stiffness and aging (51, 52). An alternative or additional mechanism involves fibrosis, whereby cytokines stimulate the production of extracellular matrix proteins like collagen and elastin, which can contribute to arterial stiffness (53, 54).

Compared to those in the L-L group of the rs3742207-TG combination, individuals at the H-H group showed a higher PWV progression. This association was more pronounced with increasing age (interaction *p* = 7.27 × 10^−09^). Notably, both the high TG groups (H-H and L-H based on rs3742207-TG combination), had essentially similar PWV progression over time regardless of risk allele (Table 2). This suggests that TG is a significant factor in the rate of PWV progression. However, both H-H and L-H groups had a significantly more PWV progression compared to the L-L group, indicating that the risk alleles of SNPs rs3742207 do partially account for an accelerated AS. Similarly, compared to those in the L-L group of the rs1495448-TG combination, individuals at the H-H group exhibited a higher PWV. The H-H group, based on the SNP rs3742207-TWBC- combination, also showed a similar pattern of association.

SNP rs3742207 is located near the *COL4A1* gene, and the *COL4A1* variant has been associated with age-related thickened intima layers and interference with endothelial function through the activation of type 2 matrix metalloprotease (MMPII), a collagenase that degrades type 4 collagen (24, 55). Agtmael et al. (56) showed that mice with mutations in *COL4A1* had defective collagen type IV in the basement membrane and altered vascular tone. Subsequently, a later study in humans reported similar findings (57). These results may reveal that collagen type IV plays a critical role in the maintenance of vascular tone, and that *COL4A1* mutations can lead to serious vascular dysfunctions. As for *MAGI1* (the gene nearest to SNP rs1495448), a variant may lead to the dysregulation of the endothelial nitric oxide synthase (eNOS) signaling pathway, resulting in reduced production of nitric oxide, a key regulator of endothelial function (58, 59).

The impact of genetic variants on AS varied with age. In both age groups, homogenous risk carriers of SNPs rs3742207 and rs1495448 risk alleles exhibited higher PWV, however, the effect was notably more prominent among the younger cohort. Our finding corroborates the findings of a previous study, highlighting that with increasing age, the influence of environmental factors becomes larger (18). These results suggest that the effect of genetic variants may be more pronounced in younger individuals, while the cumulative impact of exposure to environmental risk factors—and especially the accumulation of atherosclerosis—becomes more significant with increasing age. This observation is further supported by the stronger influence of lipids and inflammation markers within the older age group, as illustrated in Figure 3A,B. Furthermore, age-related decline in physiological function can make the body more susceptible to the damaging effects of environmental stressors. For example, in a recent study comparing the effects of genetic variation and environment on the expression of about 20,000 human genes with aging, researchers discovered that aging and environment play a much greater role than genetic variation in influencing the expression patterns of many genes as we age (60).

Our working hypothesis is that lipid and inflammation biomarkers, as well as genes *COL4A1* and *MAGI1*, partially share signaling pathways that affect the balance between relaxation and contraction of arterial vessels, leading to altered vascular function. For example, missense mutations in *COL4A1* lead to a range of vascular abnormalities including compromised endothelial cell function and dysregulation of blood pressure in animals (56). Similarly, elevated levels of TG and very-low-density lipoproteins can lead to endothelial dysfunction, which impairs the production and release of nitric oxide by eNOS. High levels of triglycerides can accumulate in endothelial cells and activate signaling pathways that reduce eNOS expression and activity (47). Besides, a large GWAS identified *MAGI1* as a significant predictor of lipid metabolism. This suggests that individuals with a risk *MAGI1* genotype and high TG levels may well experience an accelerated increase of PWV (61). In contrast, there was little concrete evidence demonstrating the significant role of *BCL11B* in eNOS signaling pathways.

A combined assessment of individuals based on their genetic, lipid, and inflammation markers can be clinically useful to identify high-risk groups for the prevention and treatment of AS, a strong predictor of CVDs and all-cause mortality. Identifying individuals at high risk for PWV can help to implement early interventions to reduce risk and potentially improve their long-term health outcomes. Additionally, this strategy can help to identify specific populations who may benefit from new therapies aimed at reducing AS and lowering the risk of CVDs. In sum, taking a comprehensive approach to assess risk factors for AS can aid in the early detection of preclinical indicators of CVDs, potentially leading to better health outcomes and improved management of these conditions.

## Strengths and limitations

One of the strengths of this study is the use of longitudinal data to assess the relationship of PWV with genetic variants, lipid, and inflammation markers over time. Unlike cross-sectional studies, this study included multiple measures of risk factors, confounding variables, and PWV which allows us to account for well-known changes over the life course that contribute to the development and progression of AS and CVDs.

One important limitation of investigating gene-environment questions related to PWV is the limited number of *a priori* SNPs that we could investigate. This reflects the relatively low heritability and the absence of alleles of very large effect, so that GWAS of much larger numbers of individuals than assessed thus far would be required to reveal more contributory alleles. Additionally, due to the limited availability of cohorts with longitudinal data on PWV, lipid, and inflammation markers, as well as genetic data, our replication possibilities were limited, which could affect the generalizability of our findings. Another limitation is the substantial dropout of study participants at each data collection phase, which may potentially lead to attrition bias and reduced power. However, our dropout analysis, showed similarities in socio-demographics, BP, lifestyle, and PWV distribution between dropouts and retained participants. Despite these limitations, our study provides valuable insights into the longitudinal relationship between PWV and genetic, lipid, and inflammation markers, and supports the goal of further research in this area.

## Conclusion

We have demonstrated that lipids (TC, LDL, TG) and inflammation markers (ESR, FGN, and TWBCs) are associated with PWV, supporting previous evidence from cross-sectional studies. Our analysis, taking into account potential confounding factors, has revealed the combined effect of risk alleles (SNPs rs3742207, rs1495448, and rs7152623) and elevated levels of lipid (TG) and inflammation (TWBCs) biomarkers in driving an accelerated rate of PWV progression over time. Further studies are required to explore whether lipids themselves or lipid metabolites are stronger predictors of AS and whether systemic or local inflammation of endothelial cells is important in the development of AS. Additionally, further studies are required to elucidate the interplay between genetic variants and environmental factors, represented by biomarkers, that can lead to structural and functional changes in arterial walls.

## Data Availability

The data will be available upon reasonable request to Professor Francesco Cucca, Department of Biomedical Sciences, University of Sasari, Italy (francesco.cucca@irgb.cnr.it) with permission from the Institute of Genetics and Biomedical Research (IRGB), National Research Council (CNR), in the SardiNIA repository link.
