# Supplementary material for: Genetic and biomarker modulation of arterial stiffness change in the SardiNIA population cohort
Source: Front Epidemiol. 2024 Jan 4;3:1295209. doi: 10.3389/fepid.2023.1295209 (PMC10910914; doi:10.3389/fepid.2023.1295209)
Supplement: Supplementary file 1 [file Datasheet1.pdf]

Supplementary Tables

Supplementary Table S1: Population characteristics of dropped outs at each follow-up time.

Characteristic	Drop out at 1st follow-up	Drop out at 2nd follow-up	Drop out at 3rd follow-up
N, total	1627	1859	1865
Socio-demography			
Age (years), mean (SD)	45.36 (19.56)	46.9 17.8	49.7 (16.9)
Sex (females), n (%)	921 (56.6)	1036 (57.0)	1062 (57.0)
Ever smoking (yes)	629 (38.7)	771 (42.4)	772 (41.4)
Ever drinking alcohol (yes)	805 (49.5)	1040 (57.2)	1060 (56.8)
Anthropometry			
Body mass index (kg/m2), mean (SD)	25.6 (5.0)	25.7 (4.7)	25.8 (4.5)
Blood pressure			
Systolic blood pressure (mm Hg), median (IQR)	127.0 (19.7)	124.6 (17.2)	124.0 (18.2)
Diastolic blood pressure (mm Hg), median (IQR)	77.2 (11.1)	77.9 (11.5)	76.0 (10.1)
Hypertension, n (%)	458 (28.1)	440 (24.8)	398 (22.5)
PWV (m/s), median (IQR)	6.5 (5.3 8.29)	6.6 (5.5 8.3)	6.6 (5.6 8.1)

Supplementary Table S2: Impact of genetic variants, lipid levels, inflammation, and the interplay between genetic variants with lipids and inflammation markers on arterial stiffness (represented by PWV).

		Lipids								
		CHO			LDL			TG		
	**Variables**	**Estimate**	**95% CI**	***p***	**Estimate**	**95% CI**	***p***	**Estimate**	**95% CI**	***p***
**SNPs**										
**rs3742207**	GG			Reference			Reference			Reference
	GT	1.013	1.005 – 1.021	**1.38E-03**	1.013	1.005 – 1.021	**1.53E-03**	1.013	1.005 – 1.021	**1.45E-03**
	TT	1.026	1.016 – 1.037	**1.04E-06**	1.026	1.016 – 1.037	**9.10E-07**	1.026	1.016 – 1.037	**7.85E-07**
	biomarker	1.006	1.00 – 1.012	5.96E-02	1.003	0.997 – 1.009	2.95E-01	1.007	1.001 – 1.012	**2.19E-02**
	biomarker*GT	1.006	0.999 – 1.014	9.19E-02	1.004	0.997 – 1.011	2.83E-01	1.004	0.997 – 1.012	2.26E-01
	biomarker*TT	1.0	0.990 – 1.009	9.31E-01	0.998	0.989 – 1.008	7.49E-01	1.005	0.995 – 1.014	3.30E-01
**rs1495448**	TT			Reference			Reference			Reference
	TG	1.004	0.996 – 1.012	3.62E-01	1.004	0.996 – 1.012	3.71E-01	1.004	0.996 – 1.012	3.41E-01
	GG	1.013	1.003 – 1.024	**1.25E-02**	1.012	1.002 – 1.022	**2.18E-02**	1.013	1.003 – 1.023	**1.38E-02**
	biomarker	1.008	1.002 – 1.014	**1.15E-02**	1.006	1.000 – 1.012	**4.02E-02**	1.005	0.999 – 1.011	1.10E-01
	biomarker*TG	1.001	0.993 – 1.008	8.40E-01	0.999	0.992 – 1.006	7.94E-01	1.004	0.996 – 1.011	3.57E-01
	biomarker*GG	1.00	0.999 – 1.009	9.23E-01	0.997	0.987– 1.006	4.66E-01	1.014	1.004 – 1.023	**6.52E-03**
**rs7152623**	GG			Reference			Reference			Reference
	GA	1.008	0.999 – 1.017	7.67E-02	1.008	0.999 – 1.017	7.96E-02	1.008	1.00 – 1.017	6.24E-02
	AA	1.017	1.007 – 1.027	**8.01E-04**	1.017	1.007 – 1.027	**8.28E-04**	1.018	1.008 – 1.028	**5.21E-04**
	biomarker	1.009	1.003 – 1.016	**5.57E-03**	1.007	1.001 – 1.014	**3.17E-02**	1.006	1.00 – 1.013	5.23E-02
	biomarker*GA	1.00	0.992 – 1.008	9.20E-01	0.998	0.990 – 1.006	6.42E-01	1.004	0.996 – 1.012	2.99E-01
	biomarker*AA	0.998	0.989 – 1.007	5.91E-01	0.995	0.989 – 1.004	3.22E-01	1.005	0.996 – 1.014	3.09E-01
		**Inflammation markers**								
**SNPs**		**TWBC**			**ESR**			**FGN**		
**rs3742207**	GG			Reference			Reference			Reference
	GT	1.012	1.004 – 1.020	**3.02E-03**	1.012	1.004 – 1.020	**2.80E-03**	1.012	1.004 – 1.020	**3.46E-03**
	TT	1.025	1.014 – 1.035	**3.74E-06**	1.026	1.015 – 1.036	**1.74E-06**	1.026	1.015 – 1.036	**1.54E-06**
	biomarker	1.006	1.001 – 1.012	**2.10E-02**	1.007	1.000 – 1.013	**3.82E-02**	1.01	1.004 – 1.016	**7.41E-04**
	biomarker*GT	1.004	0.997 – 1.011	2.48E-01	0.997	0.989 – 1.005	4.75E-01	0.996	0.989 – 1.004	3.11E-01
	biomarker*TT	1.007	0.997 – 1.016	1.68E-01	0.996	0.986 – 1.006	4.75E-01	1.003	0.994– 1.013	4.83E-01

**rs1495448**	TT			Reference			Reference			Reference
	TG	1.003	0.995– 1.012	4.03E-01	1.004	0.995– 1.012	3.86E-01	1.004	0.996 – 1.012	3.65E-01
	GG	1.013	1.002 – 1.023	**1.63E-02**	1.012	1.002 – 1.023	**1.75E-02**	1.013	1.002 – 1.023	**1.56E-02**
	biomarker	1.005	0.999 – 1.010	9.32E-02	1.009	1.003 – 1.015	**4.35E-03**	1.011	1.005 – 1.017	**1.79E-04**
	biomarker*TG	1.009	1.001 – 1.016	**1.88E-02**	0.995	0.988 – 1.003	2.20E-01	0.998	0.990 – 1.005	5.06E-01
	biomarker*GG	1.006	0.997 – 1.015	2.08E-01	0.990	0.981– 0.999	**4.38E-02**	0.996	0.986– 1.005	3.36E-01
**rs7152623**	GG			Reference			Reference			Reference
	GA	1.008	1.00 – 1.017	6.28E-02	1.008	0.999 – 1.017	6.53E-02	1.008	1.000 – 1.017	6.22E-02
	AA	1.017	1.007 – 1.027	**6.20E-04**	1.017	1.007 – 1.027	**6.40E-04**	1.018	1.008 – 1.028	**3.67E-04**
	biomarker	1.007	1.001 – 1.014	**4.71E-02**	1.009	1.002 – 1.016	**8.29E-03**	1.01	1.004 – 1.017	**2.14E-03**
	biomarker*GA	1.004	0.996 – 1.012	3.26E-01	0.997	0.989– 1.005	4.14E-01	0.997	0.989 – 1.005	4.88E-01
	biomarker*AA	1.002	0.993– 1.012	6.66E-01	0.989	0.980 – 0.999	**2.34E-02**	1.00	0.992 – 1.009	9.29E-01

Linear mixed effects model adjusted for age, sex, body mass index, diastolic blood pressure, ant-hypertension medication, plasma glucose, ever smoking, and ever drinking was used. Each SNP-biomarker pair was modeled separately. SNP: Single nucleotide polymorphism; PWV: Pulse wave velocity; TC: Total cholesterol; LDL: Low-density lipoprotein; TG: Triglyceride; TWBC: Total white blood cells; ESR: Erythrocyte sedimentation rate; FGN: Fibrinogen.

Supplementary Table S3: Effects of lipids, inflammatory biomarkers and genetic variants on arterial stiffness (represented by PWV) over time, stratified by gender.

		Males		Females	Males vs. Females
**Variables**	**Estimate (95% CI)**	***p***	**Estimate (95% CI)**	***p***	**P[b]**
**Lipids**					
CHO	1.01 (0.96-1.06)	7.80E-01	1.04 (1-1.07)	6.23E-02	0.358
LDL	0.96 (0.92-1.01)	1.26E-01	1.01 (0.98-1.05)	4.47E-01	0.121
TG	1.05 (1.0-1.1)	5.49E-02	1.1 (1.06-1.14)	**7.22E-08**	**0.005**
**Inflammation biomarkers**					
TWBC	1.1 (1.05-1.15)	**1.07E-04**	1.08 (1.05-1.12)	**6.07E-06**	0.338
ESR	1.09 (1.04-1.14)	**2.93E-04**	1.05 (1.02-1.09)	**5.16E-03**	**0.004**
FNG	1.07 (1.02-1.12)	**2.65E-03**	1.07 (1.04-1.11)	**3.85E-05**	0.613
**SNPs**					
rs3742207					
TT	Reference				
GT	1.17 (1.04-1.29)	**8.09E-03**	1.04 (0.95-1.13)	4.07-01	0.084
GG	1.29 (1.13-1.45)	**3.8E-04**	1.21 (1.08-1.33)	**9.44E-04**	0.348
rs1495448					
TT	Reference				
GT	1.09 (0.97-1.21)	1.38E-01	1.03 (0.93-1.12)	6.10E-01	0.265
GG	1.3 (1.13-1.44)	**2.66E-04**	1.05 (0.93-1.17)	4.17E-01	**0.005**
rs7152623					
GG	Reference				
GA	1.15 (1.01-1.28)	**3.08E-02**	0.99 (0.89-1.09)	8.21E-01	0.092
AA	1.23 (1.08-1.34)	**2.87E-03**	1.07 (0.95-1.19)	2.55E-01	0.13

[b]Significance level from interaction analyses between gender and the corresponding biomarkers and genetic variants. Abbreviations: PWV: Pulse wave velocity; TC: Total cholesterol; LDL: Low-density lipoprotein; TG: Triglyceride; TWBC: Total white blood cells; ESR: Erythrocyte sedimentation rate; FGN: Fibrinogen; SNP: Single nucleotide polymorphism
